# Socially engaged photography and wellbeing: reflections on a case study in the northwest of England

**DOI:** 10.1080/17571472.2018.1477439

**Published:** 2018-05-22

**Authors:** Gary Bratchford, Gina Giotaki, Liz Wewiora

**Affiliations:** a Department of Photgraphy, University of Central Lancashire, Preston, UK; b Department of Dance, Liverpool John Moores University, Liverpool, UK; c Open Eye Gallery, Liverpool, UK

**Keywords:** Socially engaged photography, wellbeing, resilience

## Abstract

This paper describes a 9-month project commissioned by Halton Clinical Commissioning Group (CCG) and Liverpool photography organisation, Open Eye Gallery. Socially engaged photographers worked with local residents from the Windmill Hill estate in Runcorn to describe healthy and unhealthy aspects of the area. Six women were trained to use cameras to document everyday things that mattered to them. Through focus groups they discussed what these photographs revealed about the health and ill-health of the area. The resulting exhibition, *As and When,* told their story. Despite being a deprived area with more than average incidence of illness, they identified many positive things that enhanced their sense of wellbeing and resilience. The benefits of the project included increased social engagement and participation, an improved sense of vitality and rejuvenation, emotional benefits, a feeling of greater political agency and increased visual literacy. This paper outlines the model of practice developed with the support of CCG and in collaboration with local stakeholders. It makes a case for the value and the ways in which clusters of general practices could develop links and work with health assets in their local communities.

## Related LJPC Papers

Michael Drucquer (2017) Three ways for art to stimulate the mind, London Journal of Primary Care, 9:4, 54-55, DOI: 10.1080/17571472.2017.1333590

Susan Hallam & Andrea Creech (2016) Can active music making promote health and well-being in older citizens? Findings of the music for life project, London Journal of Primary Care, 8:2, 21-25, DOI: 10.1080/17571472.2016.1152099

## Why this matters to me

This topic matters to me because I am passionate about the arts, communities and wellbeing. I am inspired by those who see the potential for all three to foster positive changes. To work with those who understand that each element can enrich the other in a cycle of learning, if given the right affordances, such as time, patience and the room to accommodate setbacks as ‘part’ of the learning process. The co-authoring of arts-based engagement can be insightful and empowering. It can be a resource and a tool to effect change and contribute to a greater sense of wellbeing.

## Key message

What the reader might learn from the paper (keep this as brief as possible). Socially Engaged Photography provides a way for clusters of general practices to work with health assets in their local communities.

## Introduction

“Health is influenced by the conditions in which people are born, grow, work, live, and age, and the wider set of forces and systems shaping the conditions of daily life”.

*All*-*Party Parliamentary Group Report on Arts, Health and Wellbeing: Inquiry Report* [1]

The increasing number of people who have long-term conditions has heightened the need for emphasis on prevention rather than cure in primary care [2,3]. Prevention includes a need for general practitioners (GPs) to improve the wellbeing of their populations by working with local ‘health assets’ [4–6]. An asset-based approach ‘*makes visible and values the skills, knowledge, connections and potential in a community. It promotes capacity, connectedness and social capital’* [7]. This is important for all citizens. It is especially important for those who are sick, to make them more able to contribute to self-care and shared-care [7]. A recent paper published in LJPC describes a need for public health & primary care partnerships to work together at local level to support an asset-based approach for long-term conditions. [3] This paper describes a way to do this using socially engaged photography.

In 2016, arts organisation Open Eye Gallery approached Halton CCG to co-devise and deliver one of seven socially engaged photography projects under the programme name ‘Culture Shifts’. The programme brought together professional photographers and local community groups from across the Liverpool city regions (531 local residents) to create photographic work, which reflected what mattered to them in society today. With a history of working with the idea of health as an asset, Halton CCG recognised the potential of this socially engaged photography project as a way to reveal matters that would help them to make positive policy decisions for their local context.

This paper describes the Halton CCG and Open Eye Gallery nine-month project, *As and When,* which focused on health through the process of making. In this respect, rather than representing ‘health’, photography was used as an engagement tool to bring together *and* enable a community of participants, to unpack *shared* perceptions of health and wellbeing in a site-specific exercise. In doing so, photography was a conduit for a shared experience as well as a tool to communicate. In short it addressed *and* facilitated wellbeing.

Halton CCG identified two groups to engage with the photographers for this specific project, both of which might benefit from a collaborative, arts-based project. The first was the newly formed women’s group, ‘The Women of Windmill Hill’ which is the most deprived ward in the borough of Halton. Windmill Hill has a population of 2,400 (2015) residents. Residents are 20% more likely to have cancer than in neighbouring wards. Life expectancy of men is 71 years (Halton average = 77); of women it is 77 years (Halton average = 81). Windmill Hill is in the 5% most deprived wards in the UK (DATE). [8]

The second group was The Widnes Viking RFC ‘Golden Generation’. This was a long established, 16-member group with an existing set of social activities. This group withdrew at an early stage because they felt that the project was too onerous on their time and detracted from their other activities.

## What socially-engaged photography does

Socially engaged photographers are concerned as much with processes that serve a need of the particular community of participants as with artistic outcomes. They facilitate a sense of community by engaging people in the process of taking photographs, describing the significance of what is captured and co-creating a shared narrative about their situation that leads to positive individual and collective action.

The aim of the project was to demonstrate how such engagement increases wellbeing by enhancing individual skills, improving social cohesion and developing a shared narrative that reveals how to work positively to both reduce illness and enhance health in the local area. We use the term ‘wellbeing’ to mean ‘healthy’ in the holistic sense, as described in the 1978 World Health Organisation ‘Alma Ata Declaration’ [9]. This definition goes beyond medical treatment of illness to maintain that health is everyone’s concern and initiatives are needed that help people to help themselves from which they can improve their personal action competence, social networks and employability.

Our approach to ‘wellbeing’ is also informed by the way the term has been approached in the emergent field of ‘Arts in Health’ in which this project is situated. Although, the field may not have yet established a stable self-definition, it has been primarily practice-led, existing as a set of arts-based activities taking place in various public health and clinical contexts. It is an interdisciplinary field borrowing from sociology, anthropology, ethnography and visual culture as a means to understand how socio-cultural and political determinants may affect something like wellbeing.

## The project

The project began with an open meeting in October 2016 at which interested people from Windmill Women’s Group and the Vikings Golden Generation met the artists. We intended for them to understand the project and agree whether they wanted to take part. It also allowed the photographers/facilitators to begin to understand the local context.

As a taster of the kind of activities in the project, participants took photographs of ‘barriers within their everyday environment’. This was followed by a discussion of what those barriers might be, and what they were barriers to.

**Figure 1.**
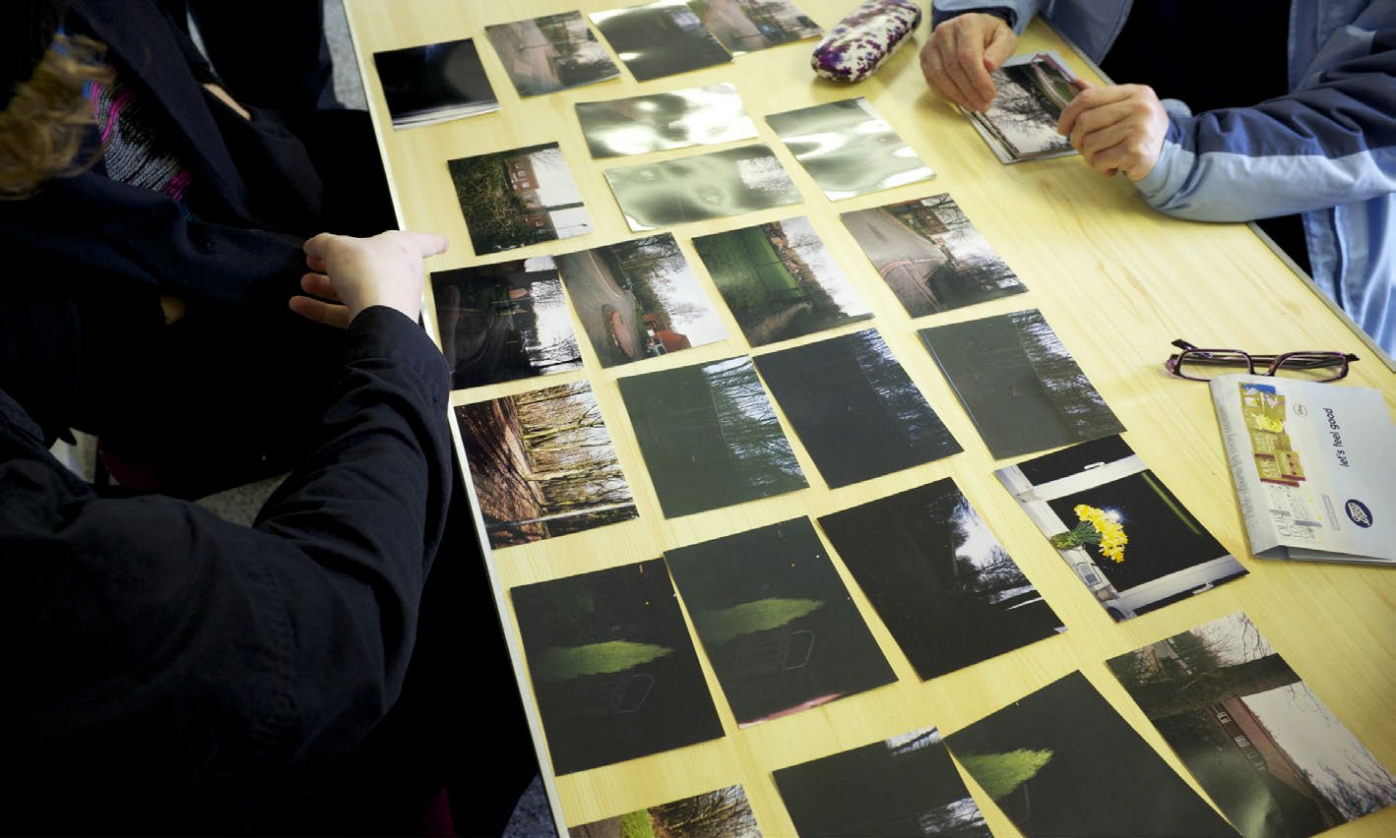
A photo from the workshops showing image analysis and photo-elicitation with a participant. The images were taken during the initial disposable camera project. We categorized them into ‘themes’ based on the content. Many of the shots were taken in the dark evenings to highlight the remoteness and isolation of the area, or from inside their houses looking out onto the dark landscape. Photo Gary Bratchford©.

We gave the participants (six women) disposable cameras to record aspects of their daily life that might indicate something about health. Each chose a selection of their photographs to discuss at follow up meetings. At meetings we asked them questions about their photographs to explore the deeper meanings they held for them. We asked about the objects in the photographs, and also things like the choice of framing, its intended meaning, what time of day it was, or how the women felt when they took the photo. The informal setting and friendly approach helped to create a playful atmosphere from which deep and broad insights into health and illness emerged. Through discussion these insights were clustered into themes (Figure 1). It was at this point, three months into the project, that the Widnes Vikings Golden Generation Group decided to opt out of the project because of the demands on their time.

Using the disposable cameras, the Women of Windmill Hill went on photo walks, undertook writing exercises and reviewed archive photographs, to explore issues that may not have otherwise been considered [10]. Through cycles of taking photographs, discussing their meaning and group discussions a narrative emerged about how the women felt about being residents on the estate. This narrative provided background for the photography exhibitions in which the project culminated.

## Results

The process turned the women photographers into ‘active lookers’. When prompted to explore a space with purpose, each participant photographed things that were important to her. The photographs stimulated conversations about spaces, places and environments that impacted on their health.

The narrative created by the participants showed that their sense of wellbeing was closely linked to their environment. Developing this narrative uncovered positive and negative aspects of the women’s environment and ways in which this impacted on them. The encouragement and guidance offered to creatively explore their local area, capture things of significance and reflect on content developed participants’ familiarity and sense of belonging in their environment. As well as developing greater visual literacy, the women felt better able to move around their area with purpose. By exploring their environment more critically, they sought out new routes and in turn, becoming more physically and cognitively active. The project enabled the women to better articulate their concerns and feelings, a common outcome of photo-driven participatory projects [11]. It led to them feeling ‘socially engaged’ – duty bound to highlight their environment for positive change and use their photos to open dialogue with local authorities.

The narrative also had negative elements. For example, the poorly lit and narrow design of the estate made some feel unconfident to move around it, especially at night. Some did not feel safe in the surrounding woodland. Others felt socially isolated as a result of the estate’s design and their lack of amenities. Surrounded by woodland, the estate is serviced by one local shop and fish and chip shop. The only way off the estate into the main town, aside from a car is by bus that stops at 6:30 pm each night.

**Figure 2.**
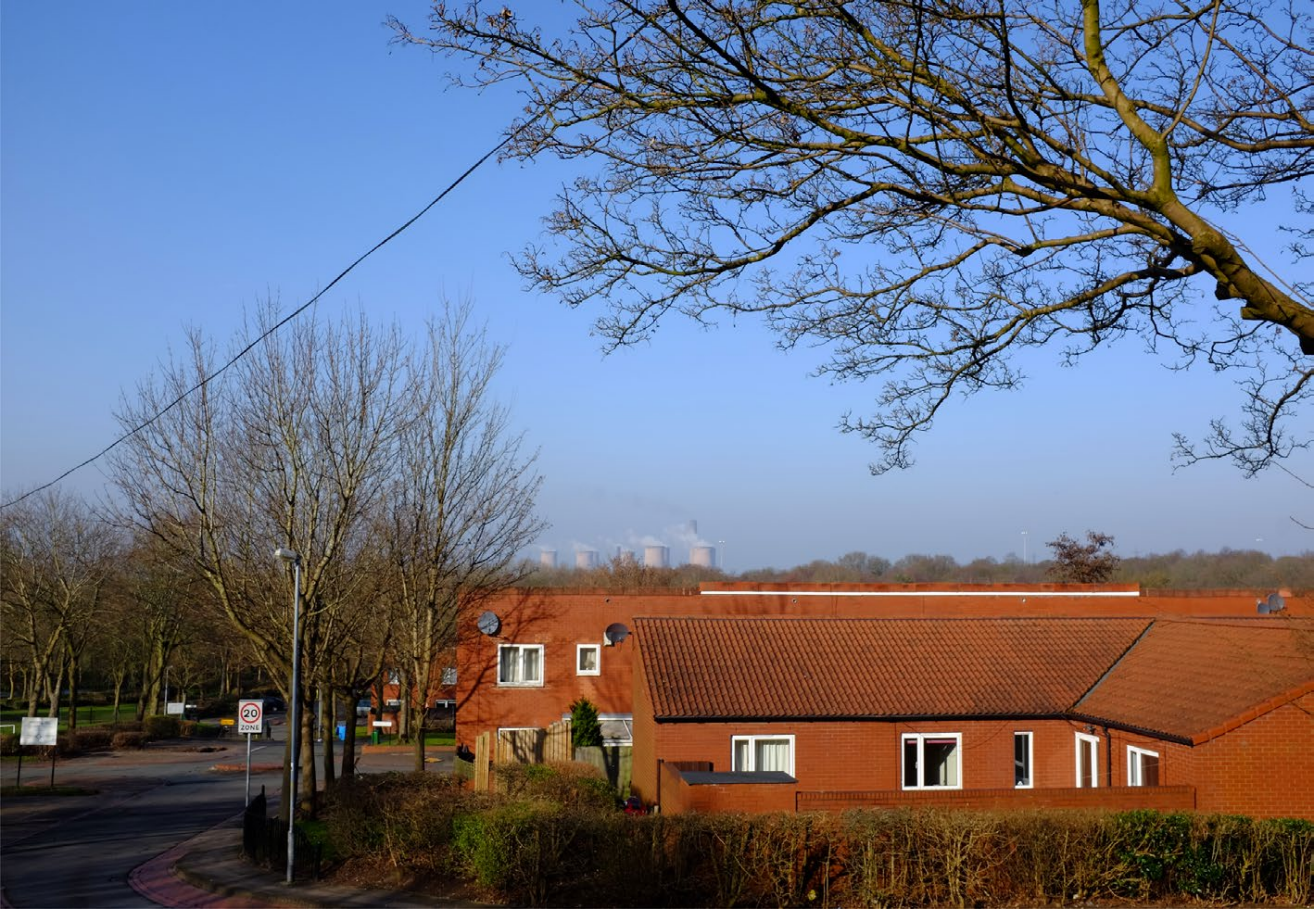
Windmill Hill is a densely built 1970s housing estate with pockets of woodland running through the estate, causing a discontinuity in the environment. This dark woodland is juxtaposed with tightly packed, dark, red brick houses. The uniformity of the planning reduces the sensorial texture of the space. In the background you can see the power station. A structure that can be seen from almost any vantage. This photo was taken from the top of the estate, which sits on a gentle slope. elderly residents at the bottom of the estate struggle to reach the shops, situated at the top of the estate due to the gradient. Photo: Gary Bratchford©.

Participant reflections highlighted already known facts about the area, including the lack of opportunities, the perceived detachment of the estate from neighbouring communities and the architecture of the estate; a physical and oppressively dense and darkly built red-brick environment (Figure 2), high unemployment and social mobility (33% of the working population are jobless and claiming benefits).

Participant reflections also highlighted things that could be done to improve wellbeing. The project helped to put in place the foundations for a women’s only photography group, the *Windmill Hill Snapper* and highlighted the benefits of photography as a means of democratic communication.

The project became well known, through local radio coverage and two well attended photography exhibitions. Though these outcomes are important, it is the less visible aspects of the project that have had the greatest legacy. Wellbeing came from participation in the process of taking the photographs and then reflecting on them to co-create a shared narrative that was interesting and understandable to outsiders.

**Figure 3.**
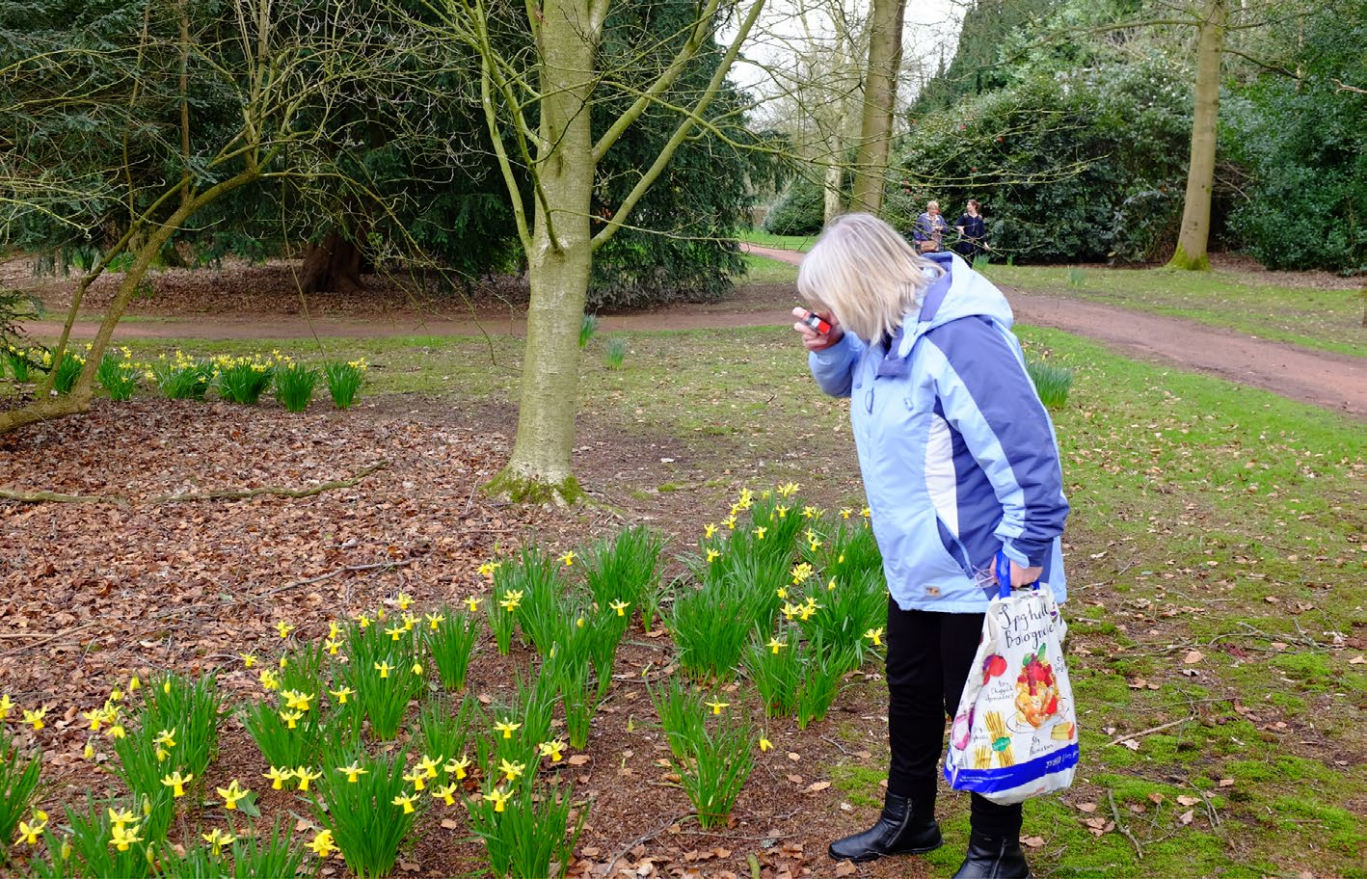
On a photo-walk, a participant takes the artists through the surrounding woodland. Photo Gary Bratchford©.

Wellbeing was improved for participants *through* the project:^Figure 3.^

• Undertaking photo walks, the women felt invigorated (Figure 3). The ability to take the women to spaces where they felt uncomfortable, such as into the woodland and surrounding green space developed a confidence to explore these spaces.
• The coming together of like-minded individuals who previously had never met created a new, supportive, social network which in turn became a self-constituted photography group that now meets on a regular basis. It bids for money to access new spaces, goes on trips and buys new equipment.
• Taking part in the process helped the women photographers to better appreciate their environment.
• A sense of empowerment was developed that enabled them to become active members of ‘their community’ [Windmill Hill] and as a community group within the estate [Windmill Snappers] all evidenced in part by the following quotes:

I took these [photos] because I could

I didn’t feel self-conscious [to look] when I had the camera

The camera helps you better see the area where you live

The project did more that develop participants’ creative skills and provide a narrative useful to the CCG. It provided a creative outlet, an opportunity to build relationships through collaborative working, a sense of belonging and a reason to think positively about their future and empowered to look at their environment in an engaged way. The women felt more active. They felt they had purpose, coupled with increased mobility, improved vitality and sense of rejuvenation. In doing all this, the project contributed to wellbeing.

### We observed three weaknesses of the project

(1) The nine months allocated to the project was not enough. Relationships and skills to engage took time to build, and misunderstandings and uncertainties took time to resolve.
(2) The coupling of the project with the CCG and an Arts institution impressed upon participants a pressure that to some degree shaped and defined the nature and pace of the work. Due to the time limitation and the use of a gallery, concerns were raised about the reception of their work in a formal setting. Most of the women had never attended a gallery before and had preconceived notions about the social and cultural divide of the audience and their sense of belonging to such spaces. Moreover, as ‘amateur works’ concerns were also raised as to the reception of their work in terms of its quality. While these issues were overcome, and as the outcomes addresses, it was a positive experience, the scheduling and introduction of formal spaces need to be mutually agreed upon rather than imposed.
(3) Our approach to evaluation was inadequate to capture the array of empowering outcomes that happened.

## Discussion

Community development initiatives linked to general practice were developed even before the NHS started, as ways to promote health, independence and access to local services. Examples include the Peckham experiment, Kark’s Community-Oriented Primary Care [12] and Ashton’s New Public Health [13]. The Bromley by Bow Centre was cited as an example in the 2006 White Paper Our Health, Our Care, Our Say [14]. The objectives of such a project were to encourage a focus on prevention and wellbeing, patient-centred care, and better integration of services. It highlighted the role of the third sector in delivering services that promote wellbeing and an ultimate impact to reduce pressure on GP services. Each of these initiatives included a long-term strategy to work with local people to both treat illnesses and promote health for everyone in the area. They combined the functions of primary care and public health.

Our experience of the Windmill Hill project and the other six projects in the *Culture Shifts Programme* shows that socially engaged photography can be a valuable mechanism to develop local health communities. It has the potential to increase wellbeing by enhancing individual skills, improving social cohesion and developing a shared narrative that helps local people to collaborate for positive action for health. In the case of Windmill Hill, it enabled us to present the local story in ways that participants felt to be authentic and at the same time develop new relationships and skills that improved their sense of wellbeing. The project shows that community-oriented and tailored, issue-based artistic practice, in this case socially engaged photography, can both reveal and develop health assets in a community.

As Dave Sweeney, Deputy Chief Officer of Halton CCG (2017) argues, the approach is particularly well-suited to help CCGs understand local health needs and health assets in ways that are well accepted.

Since Culture Shifts we have continued to develop this way of working, we have tried and tested this across Halton, in our community sector and around Well North. It’s gone down an absolute storm

How we evaluate community development projects like these can be a difficult question. The dominant approach to evaluation, from the positivist school, expects outcomes to happen as predictable consequences of purposeful action. Outcomes from community development projects like this do not happen in this direct way. Instead they come from the ways in which the process empowers people to do things for themselves. This can lead to long-term and unexpected outcomes. Evaluation approaches from the schools of critical theory and constructivism are better able to deal with this complexity. To evaluate this kind of project we need to develop an approach that can use insights from each of these schools of thought. This will help to accommodate the diversity of expectation that comes when there are multiple partners, stakeholders, audiences and pre-determined assessment points like public exhibitions in formal ‘white cubes’ settings. For Frogget et al (2011) the notion that ‘cultural organisations and the art they commission/produce [might] bring about change in individuals and communities’ has become a key text for organisations and socially engaged practice in a broad sense. Frogget’s work unpacks the relationships and potential value for participants engaging in arts-based practices supported by cultural institutions. Focusing specifically on the North of England, longitudinal fieldwork with four key institutions and empirical data, Frogget suggests that ‘Individuals and communities who participate in socially engaged art acquire new languages of social awareness – thereby expanding the possibilities of experiencing and representing the world differently’. [15].

Guba and Lincoln argue that ‘4th Generation Evaluation’ [16] has the power to combine insights from positivism, critical theory and constructivism. It uses methods from each paradigm to generate data. At a sequence of stages stakeholder groups critique the data in the light of their own experiences and from this shape the next stages of the emerging story. It is a form of participatory action research. [17]

The 2015 *Connected Communities Project* [18] describes outcomes that community development projects often produce. This helps to inform specific measures that such projects could use. The Connected Communities Project identified four different kinds of health outcomes from community development interventions:

(a) *A wellbeing dividend*. Social connectedness correlates strongly with wellbeing
(b) *A citizenship dividend*. Empowered citizens can improve their own health
(c) *A capacity dividend*. Networks and relationships can positively affect health
(d) *An economic dividend*. Capacity building and enhanced social relationships enhance employability prospects, and therefore contributes to health and welfare savings.

There are other methodological tools, drawn from the humanities, with a focus on qualitative data, that evaluate the effects of community development initiatives, including the effect of participatory arts projects with attention paid to indirect and multi-dimensional outcomes.[19–21] To evaluate the impact of collaborations between arts groups and clusters of general practices we need to work with others in the field of community empowerment to develop a set of instruments that will provide us with reliable evaluation reflective of all potential effects of a project.

The skills to lead this work go beyond technical aspects of taking good photographs. Leaders need to facilitate group process and know how to develop wellbeing in the broadest sense. Leaders need to be resourceful team-players, system-thinkers and community-developers who can use the creative process to develop these skills in the participants. As Stevie Bezencenet notes (1986) the ‘*skills which are necessary to community projects using photography are not only visual ones* – *the production of the image may be only part of the process, which may involve collective debate and authorship, research and writing… organisation and campaigning, and always, a consideration of the audience and how they will be able to interrelate with the work.*’[22]

In socially-engaged photography, the photographs are both a medium through which artists and health practitioners can generate data, and also a tool for participants to ‘speak back’. Speaking back is more than simply ‘giving voice’. It also includes a space and process for participants to formulate their ideas then take them to a range of sectors, thereby enhancing visibility of personal and community narratives. In this project, this included two gallery exhibitions, publications and radio interviews.

The lengthy nature of process can be an obstacle to developing this kind of work at scale. However, this might be dramatically shortened if participants were already familiar with the processes. We therefore propose the need for an annual programme of such projects, led by various community-developing disciplines, not merely photography. When the system embeds such activities as a routine, it can happen much more quickly and carry trust over from one project to another.

Infusing communities with lasting, high quality creative and participatory activities, led by artists specialised in socially engaged, ‘applied’ or ‘community-based’ approaches will develop specialist skills and creative thinking as well as an array of other social and personal benefits for the community. Amongst the various community-developing disciplines, the participatory arts have a capacity to identify individuals as assets in the community and work with them to re-activate the social fabric, strengthen resilience and enhance wellbeing of communities and individuals. The primary value of the work produced lies in the way cultural organisations and medical providers seek to facilitate positive outcomes through creative practices and the opportunities they provide. By promoting wellbeing through the commissioning of such projects, participants can, through co-authorship, develop connections between the general and the particular or themselves and their communities in ways that are enriching and enabling.

This paper has shown that socially-engaged photography can be used, at scale, to help people in different geographic areas work with primary care practitioners to identify local health assets and improve the health of the local population. Unlike more top-down approaches, it allows local people to ‘hold the camera and tell the story’. As we begin to reframe how patients can shift from being service users to being active participants in health and care we must consider ways that a sequence of such co-creative initiatives can be embedded within the new NHS.

For a more detailed account of the project, please see: *As and When: Documenting Socially Engaged Practice* (Bratchford and Parkinson 2017). Information and reflections on the entire *Culture Shifts* programme will be published later in 2018.

Governance. Open Eye Gallery oversaw this work with support from LJPC editor.

## Disclosure statement

No potential conflict of interest was reported by the authors.
